# Extracellular Vesicles: A New Prospective in Crosstalk between Microenvironment and Stem Cells in Hematological Malignancies

**DOI:** 10.1155/2018/9863194

**Published:** 2018-05-27

**Authors:** Ilaria Laurenzana, Daniela Lamorte, Stefania Trino, Luciana De Luca, Concetta Ambrosino, Pietro Zoppoli, Vitalba Ruggieri, Luigi Del Vecchio, Pellegrino Musto, Antonella Caivano, Geppino Falco

**Affiliations:** ^1^Laboratory of Preclinical and Translational Research, IRCCS-Referral Cancer Center of Basilicata (CROB), Rionero in Vulture, Italy; ^2^Department of Science and Technology, University of Sannio, Benevento, Italy; ^3^CEINGE-Biotecnologie Avanzate s.c. a.r.l., Naples, Italy; ^4^Department of Molecular Medicine and Medical Biotechnology, Federico II University, Naples, Italy; ^5^Scientific Direction, IRCCS-CROB, Rionero in Vulture, Italy; ^6^Section of Stem Cell and Development, Istituto di Ricerche Genetiche “Gaetano Salvatore” Biogem s.c. a.r.l., Ariano Irpino, Italy; ^7^Department of Biology, University of Naples Federico II, Naples, Italy

## Abstract

The bone marrow (BM) microenvironment in hematological malignancies (HMs) comprises heterogeneous populations of neoplastic and nonneoplastic cells. Cancer stem cells (CSCs), neoplastic cells, hematopoietic stem cells (HSCs), and mesenchymal stromal/stem cells (MSCs) are all components of this microenvironment. CSCs are the HM initiators and are associated with neoplastic growth and drug resistance, while HSCs are able to reconstitute the entire hematopoietic system; finally, MSCs actively support hematopoiesis. In some HMs, CSCs and neoplastic cells compromise the normal development of HSCs and perturb BM-MSCs. In response, “reprogrammed” MSCs generate a favorable environment to support neoplastic cells. Extracellular vesicles (EVs) are an important cell-to-cell communication type in physiological and pathological conditions. In particular, in HMs, EV secretion participates to unidirectional and bidirectional interactions between neoplastic cells and BM cells. The transfer of EV molecular cargo triggers different responses in target cells; in particular, malignant EVs modify the BM environment in favor of neoplastic cells at the expense of normal HSCs, by interfering with antineoplastic immunity and participating in resistance to treatment. Here, we review the role of EVs in BM cell communication in physiological conditions and in HMs, focusing on the effects of BM niche EVs on HSCs and MSCs.

## 1. Introduction

Normal hematopoietic stem cells (HSCs) reside in bone marrow (BM) and are supported by specialized and strictly organized stem cell niches, like endosteal and vascular [1]. The communication with other BM cells, including mesenchymal stromal/stem cells (MSCs), is crucial for HSC self-renewal, survival, and behavior. This dialogue within BM cell populations takes place through numerous extracellular and intracellular factors including hematopoietic growth factors and their receptors, signaling pathways, and cell cycle signaling [2].

Genetic alterations in HSCs or progenitors are associated to several hematologic malignancies (HMs) such as myelodysplastic syndrome (MDS), myeloproliferative neoplasia, acute myeloid leukemia (AML), chronic myeloid leukemia (CML), chronic lymphocytic leukemia (CLL), and acute lymphoblastic leukemia [3]. Following genetic alterations, HSCs or progenitors are transformed into leukemia stem cells (LSCs) that retain self-renewal capability and uncontrolled differentiation into leukemic blasts [4]. LSCs reside in the same niche as healthy HSCs and, on one side, they benefit from BM niche support and, on the other side, they modify the BM niche in order to induce a favorable environment for leukemic growth hampering normal hematopoiesis [5]. In addition, the interactions between LSCs and the endosteal niche sustain their silent state and protect them from the cytotoxicity of conventional chemotherapy [6, 7].

Studying the crosstalk between HSCs, LSCs, hematological neoplastic cells, and the BM microenvironment will enhance our comprehension of some human diseases including several HMs and the discovery of new potential therapies.

Extracellular vesicles (EVs) are emerging as new players in the intercellular communication and as new potential biomarkers for diagnosis and prognosis of human diseases [8–12]. They are a heterogeneous group of cell-derived vesicles including exosomes (Exo) and microvesicles (MVs) with a size ranging between 15 nm and 10 *μ*m in diameter and with diverse biogenesis [13]. Different cells in physiological and pathological conditions, including tumor cells, can secrete EVs [14]. They act both in short-range intercellular communication, for example in the medullary microenvironment or in coculture conditions, and in long-range communication when released into the bloodstream through which they can reach secondary sites and give rise to premetastatic niches [15–17]. EVs carry part of DNA, RNA, proteins, lipids, and metabolites of the origin cells. Since EVs are present in biological fluids such as blood, urine, and sperm, [18, 19] and are a representative part of the whole cell for their phenotype and content, they could be used as a diagnostic tool by mimicking a “liquid biopsy.” These last characteristics make them excellent candidates as diagnostic and/or prognostic biomarkers in different diseases, especially in tumors, through noninvasive or minimally invasive procedures. In our recent study, we found that serum EV number and their specific *oncomiRNA155* are higher in HM patients than in healthy subjects and, more importantly, EVs exposed specific tumor-associated surface markers [20, 21].

Stem cells (SCs) from embryos [22, 23], from different adult tissues such as BM, liver, and adipose tissue, and from induced pluripotent SCs, release EVs [24, 25]. Moreover, embryonic SC-EVs deliver mRNAs of pluripotent transcriptional factors such as HoxB4, Nanog, Oct3/4, and Rex-1, and transfer them to recipient cells, supporting hematopoietic progenitor cell expansion [26]. In addition, SC-EV microRNAs (miRNA) downregulate cell adhesion molecule levels, contributing to hematopoietic progenitor cell mobilization [27]. In a tumor context, SCs secrete EVs, which act as a means of communication in the tumor microenvironment playing multiple roles in tumorigenesis, and both in tumor angiogenesis and metastasis [28]. Finally, in *in vivo* models, SC-EVs mainly exhibit an inhibitory effect on the immune system suppressing proinflammatory processes and reducing oxidative stress and fibrosis [29]. Remarkably, MSC-EVs promote tissue renewal by inducing a proregenerative environment allowing stem and progenitor cells to successfully maintain tissue homeostasis. Importantly, MSC-EVs were used in two human disease therapies. In the first study, the administration of MSC-EVs reduces graft-versus-host disease (GvHD) symptoms and reduces steroid doses in an allogeneic transplantation of patients suffering from steroid refractory GvHD [30]. In the second study, the MSC-EV therapy triggers the regeneration within the affected kidney in patients with chronic kidney disease [31].

Although much has been reported about the stem cell and MSC-EV role, less is known about the influence of BM-EVs on HSCs and MSCs in physiological conditions and in malignancy onset, progression, and therapy resistance. In this review, therefore, we will discuss the recent advances in the field of EVs as actors in communication between cells within the BM niche in physiological conditions and in HMs, underlining the role and the effects in the tumor microenvironment-stem cell crosstalk. In particular, we will focus on the effects of EVs from BM niche cells on HSCs and MSCs.

## 2. Stem Cells

### 2.1. Hematopoietic Stem Cells (HSCs)

HSCs are the only cells into the hematopoietic system that possess the potential for both pluripotency and self-renewal [1]. Pluripotency is the ability to differentiate into all functional blood cells; self-renewal is the ability to generate identical daughter cells without differentiation [32]. Postnatally, the BM is the primary site of HSC maintenance and hematopoiesis, but hematopoietic stress reallocates the niche to the spleen and induces extramedullary hematopoiesis. Although HSCs comprise only about 0.005–0.01% of the BM cell population, each single HSC retains the capability alone to reconstitute the entire hematopoietic system [33].

In AML, leukemia initiating cells (also named LSCs) represent a rare cell population that self-renews and generates an immature progeny invading and perturbing normal hematopoietic tissues [34]. HSCs and LSCs physically and functionally interact with the BM niche [35]. It is demonstrated that both HSCs and LSCs can be extended *in vitro* for a long time either in environmental conditions that mimic BM support or in coculture with BM stromal cells. These observations reinforce the crucial role that the BM niche, in particular the stroma, plays in healthy and leukemic stem cell homeostasis [5, 36–38]. It is still controversial whether cell-cell contact between hematopoietic stem/progenitor cells (HSPCs) and stromal cells is necessary to promote the hematopoietic cell expansion [39–43]; it is indeed clear that the definition of niche components and how they regulate hematopoiesis will provide the opportunity to improve regeneration after injury or HSC transplantation and to understand how disordered niche function could contribute to diseases, in particular to HMs.

### 2.2. Mesenchymal Stromal/Stem Cells (MSCs)

The International Society for Cellular Therapy reported the minimal criteria for MSC definition: (i) they adhere to plastic under standard culture conditions; (ii) they express CD73, CD90, and CD105; (iii) they lack the expression of CD45, CD34, CD11b or CD14, CD19 or CD79a, and HLA-DR; and (iv) they have the potential to differentiate into the osteogenic, chondrogenic, and adipogenic cell lineages [44, 45]. MSCs may be isolated from BM, umbilical cord, liver, adipose tissue, and multiple dental tissues [46, 47]; here, we will focus on MSCs derived from BM. They maintain long-term, quiescent HSCs through the presentation of surface signals and the secretion of major stemness supportive cytokines such as leukemia inhibitor factor and IL-6 [48, 49].

On the contrary, MSCs from leukemic patients hamper *in vitro* hematopoietic cell expansion and differentiation. In particular, AML-patient MSCs significantly impair the expansion of human umbilical cord blood CD34^+^ progenitors and limit their differentiation to maintain a stable pool of immature quiescent precursors (CD34^+^ CD38^−^) compared to healthy donor-derived MSCs (hereafter healthy MSCs) [50]. Remarkably, healthy MSCs maintain AML patient blasts in a quiescent state resulting in increased leukemic survival after treatment with cytarabine [51]. Overall, MSCs have a functional role in the regulation of the BM microenvironment, in particular by influencing the immune system and angiogenesis and in supporting hematopoiesis [52–55] and, consequently, they are widely used in allogeneic hematopoietic stem cell transplantation [56, 57].

However, much work remains in defining the relationship between MSCs, HSCs, and other niche cells, especially on how they interact with each other and how these interactions regulate the hematopoiesis. Uncovering how the microenvironment participates in normal and HM progression will enhance new approaches to hematological disorders.

## 3. Extracellular Vesicles

On the basis of biophysical properties (i.e., size and shape) and the mechanism of biogenesis, EVs are classified into Exo, MVs, apoptotic bodies, and oncosomes [58, 59].

Exo are the smallest EVs (20–150 nm) that are generated inside multivesicular bodies which are secreted after their fusion with the plasma membrane [60, 61]. They show a higher rigidity of their lipid bilayer compared with that of cell membranes, making them resistant to degradation and useful as vehicle of different biomolecules. The formation and the release of Exo take place through both endosomal sorting complex required for transport-dependent or -independent mechanisms [60, 61].

MVs enclose EVs with a more heterogeneous size (50–1000 nm) bud directly from the plasma membrane and, for this reason, their surface markers are largely dependent on the composition of the membrane from which they derive [59].

Apoptotic bodies are membrane blebs that are released during cell apoptosis [62] with a diameter ranging between 50 nm and 5 *μ*m, contain DNA binding histones, and are depleted in glycoproteins [63, 64].

Lastly, oncosomes are the largest EVs (1–10 *μ*m in size) produced by membrane protrusions of malignant cells that lug bioactive molecules involved in the progression of cancer [64, 65].

The release of EVs from donor cells can be constitutive or be induced in response to activation or stress signals [64], including glucose and intracellular Ca^2+^ concentrations, oxygen tension, and microenvironmental pH [66]. Interestingly, EVs contain cargos of diverse nature including nucleic acids (i.e., mRNA, noncoding RNA such as miRNA, transferRNA, and genomic and mitochondrial DNA), cytosolic and membrane proteins, lipids, cellular organelles like mitochondria [67, 68], and metabolites [69, 70]. Interestingly, some databases such as EVpedia, Vesiclepedia, and ExoCarta collect the currently known components of EVs [71–73].

Notwithstanding, the content of EVs generally reflects the nature and the status of the donor cell: EVs could be enriched or depleted of specific materials with respect to origin cells [64, 74]. Likewise, EV cargo nature and abundance are also influenced by the pathways that lead to the formation of different EV subtypes [75].

The total cargo of human MSC-EVs is recently defined by next generation sequencing and proteomic analyses. They are enriched in proteins that support tumor (PDGFR-*β*, TIMP-1, and TIMP-2), lipids (sphingomyelin and diacyl-glycerol), metabolites (glutamic and lactic acid), several oncomiRNAs (*miRNA21* and *miRNA34a*) [76], critical surface markers, and signalling molecules characteristic of MSCs [77]. A recent work reports that BM-MSC-Exo are highly enriched in transferRNAs that represent more than 35% of the total small RNAs, while miRNAs account for only 2–5% [78]. This composition differs in MSC-Exo released from other tissues. In addition, BM-MSC-EVs contain a pattern of miRNAs essential for the metabolism, proliferation, differentiation, and homing of SCs [79]. Additionally, different chemokines, such as MCP-1, IP-10, and SDF-1, are found in BM-MSC-Exo in multiple myeloma (MM) [62]. These chemokines are important in supporting MM cell viability.

### 3.1. EV Uptake from Recipient Cells

Once released, EVs reach recipient cells where they exert pleiotropic effects through distinct signalling cascades via autocrine, paracrine, and juxtacrine feedback loops [80].

EVs can be internalized into recipient cells with different mechanisms including endocytosis, direct cell surface membrane fusion, and a lipid raft-mediated energy-dependent process, or they can remain permanently associated with plasma membrane [81].

Surface molecules, such as integrins or receptors, and microenvironment conditions control the EV uptake by regulating their specific cell tropisms, while EV cargo, released into target cells, alters their composition by inducing phenotypic, functional, and epigenetic modifications [17, 82].

In particular, the specific integrin-mediated adhesion of tumor Exo to specific cell types and organs induces the metastatic niche formation [83]. Similarly, BM dendritic cell Exo are preferentially internalized by splenic conventional dendritic cells, rather than by B-lymphocytes, macrophages, or splenic T cells [84]. Moreover, Exo from mantle cell lymphoma cells are preferentially taken up by themselves while only a minor fraction of Exo was internalized into T-cell leukemia and BM stroma cell lines [85]. The specific cell type uptake of EVs has also been observed *in vivo*. In fact, human MSC-EVs injected into the blood stream of mice primarily accumulated in the liver, spleen, and in sites of acute kidney injury, where they facilitated injury recovery [86]. Similarly, melanoma-derived Exo accumulated in the lungs, bone, liver, and spleen and they increased the frequency of metastasis at these sites [87]. Finally, Parolini et al. reported that low pH favors Exo uptake by melanoma cells [88].

## 4. Role of EVs in Physiological BM Niche

As reported, MSCs are commonly studied as EV donor cells. EVs from BM-MSCs shuttle the selected molecular cargo to recipient cells targeting genes involved in organogenesis, cell survival and differentiation, tissue regeneration, immunomodulation, and angiogenesis [79, 89–91]. Nevertheless, the role of MSC as EV target cells must not be ignored. In fact, EVs derived from differentiated cells are able to modulate the MSC phenotype [92]. In particular, miRNA contained in EVs released from neuronal [93], endothelial [94–96], and kidney epithelial [97] cells induce proliferation, migration, and secretion of soluble factors in MSCs.

Immune cells, such as monocytes, use EVs to communicate with MSCs modulating their phenotype by upregulating osteogenic gene expression [98]. In fact, Ekström et al. demonstrated that both RUNX2 and BMP-2 expression is significantly increased in MSCs after monocyte-EV stimulation, whereas no significant difference is observed in osteocalcin [99], an osteoblastic gene regulated by BMP-2 via RUNX2 [100].

Regarding the hematopoietic system, Ratajczak et al. demonstrated that, besides coagulation, MVs derived from activated platelets play a role in important biological processes. In particular, these last enhance the chemotactic responsiveness of HSPCs, and increase their survival and proliferation by transferring specific mRNA and proteins [101]. In another study, the same authors reported that EVs released from embryonic SCs sustain HSPC stemness and multipotency by delivering specific “stemness” mRNAs [101].

More recently, it was demonstrated that mRNA and miRNA in mast cell EVs have been transferred to CD34^+^ progenitors. In fact, Ekström et al. identified, by using miRNA microarray analysis, 116 miRNAs in Exo and 134 in donor mast cells. Furthermore, microarray experiments revealed the presence of approximately 1800 mRNA in Exo, which represent 15% of the donor cell mRNA content. Transfer experiments reveal that Exo could shuttle RNA between human mast cells and CD34^+^ hematopoietic progenitor cells suggesting their role in cell communication [102].

A recent discovery showed that stromal cells release biologically active EVs which act on HSPCs. Specifically, two murine stromal cell lines, one with and the other without HSPC supportive capacity, produce different EV types in terms of size and of small RNA and mRNA signature. Lin^−^Sca1^+^cKit^+^-HSPCs preferentially take up EVs produced by a supportive stromal line (suppEVs) but not those released by a nonsupportive one. SuppEVs transfer mRNA and miRNA in Lin^−^Sca1^+^cKit^+^-HSPCs by modifying their gene expression profile. Importantly, suppEVs maintain the survival and clonogenic potential of Lin^−^Sca1^+^cKit^+^-HSPC by inhibiting their apoptosis [103]. Collectively, these data assert that EVs constitute an important novel communication system in mediating the HSPC-supporting capacity of MSCs.

## 5. EV Role in BM Niche of Hematological Malignancies

It is now clear that BM cell populations, including malignant cells, influence the tumor microenvironment, via autocrine [104] or paracrine mechanisms through the secretion of soluble factors including EVs [105]. In HMs, neoplastic EVs promote tumor progression via an autocrine loop which includes interacting with their producing malignant cells, supporting autosustainability, and increasing aggressiveness [58, 105]. This relevant cross-talk mechanism is clearly demonstrated in MM [106], in pre-B acute lymphoblastic leukemia [107], in erythromyeloblastoid, and CML [108].

EVs from resistant neoplastic cells can transfer drug resistance to sensitive cells in AML [109, 110]. In particular, EVs from apoptosis-resistant AML cells modulate the expression of apoptosis-associated proteins in chemotherapy sensitive blasts [109]. A multiresistant AML cell line transfers, through EVs, chemoresistance to sensitive acute promyelocytic leukemia cells [110].

BM-MSC derived EVs induce survival, proliferation, and migration of MM cells *in vitro* and *in vivo* in a mouse model [111, 112]. Finally, Exo from AML MSCs and not from healthy MSCs protected a leukemic cell line carrying FLT3 internal tandem duplication from treatment with a specific FLT3 inhibitor [113].

**Figure 1:**
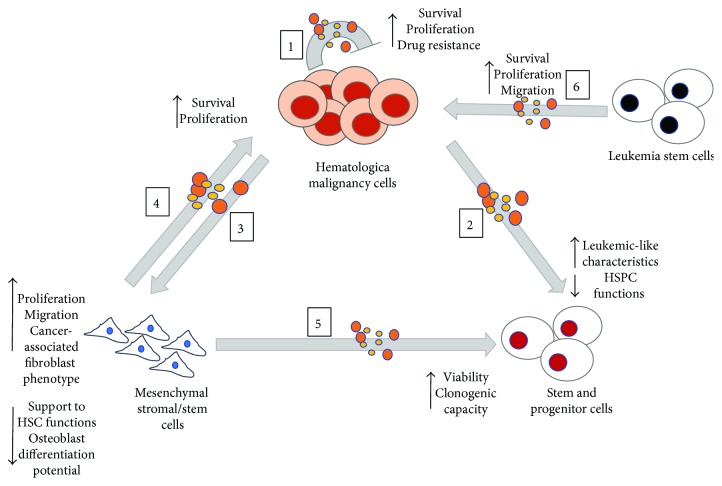
A schematic drawing of neoplasm EV effects in BM of HMs. Tumor EVs (colored balls) can (1) render malignancy more aggressive through autocrine mechanisms via (2) the induction of a suppression of hematopoietic stem/progenitor cell (HSPC) functions and a stem cell malignant transformation and (3) modification of mesenchymal stromal/stem cells (MSCs) reducing their HSC support. On the other hand, “reprogrammed” MSCs release EVs that (4) support the proliferation of malignancy cell proliferation and (5) promote HSPC viability and clonogenicity. In addition, leukemia stem cell EVs induce proliferation and migration of malignant cells (6). Arrows turned upwards (∧) and downwards (∨) indicate an increase and a reduction, respectively.

HM-EVs exert also the immune modulation effects; malignant EVs inhibit natural killer cell cytotoxicity, promote T cell apoptosis, and enhance immunosuppressive activity of myeloid-derived suppressor cells *in vitro* and *in vivo*. These EV effects are reported in B and T cell lymphomas [114, 115], CLL [116], AML [117], and MM [62, 118, 119]. Overall these data support the idea that there is indeed a complex and intriguing EV-mediated cross talk between malignant cells and BM cells that defines a favorable neoplastic microenvironment. In this context, we summarize the role of HM niche EVs on SCs and MSCs in Figure 1.

### 5.1. HM Niche EVs versus SCs

Different studies reported that Exo released from AML cell lines impair hematopoiesis by suppressing HSPC clonogenicity and by reprogramming stroma [120, 121]. According to Razmkhah et al., BM-AML-MVs promote the survival of healthy HSCs by inducing leukemic molecular characteristics, like high level of *miRNA21* and *miRNA29* [122]. Interestingly, an essential role of VPS33B in Exo pathways in HSCs and in leukemia development at early stage was demonstrated. In fact, its deletion in an *in vivo* AML model impairs the maturation and secretion of Exo and delays the AML onset [123]. Interestingly, MVs released from LSCs enhance proliferation, migration, and inhibition of apoptosis of AML cells. LSC-MVs containing a high level of *miRNA34a* inhibits the effects of LSCs on AML cells [124, 125].

Muntion et al. suggested that MVs derived from MSCs of MDS patients modify CD34^+^ cell properties, promoting their cell viability and clonogenic capacity and altering their miRNA and gene expression profiling [126].

EVs released by myeloproliferative neoplastic MSCs, enriched in *miRNA155*, induce an increase of granulocyte colony forming unit number in neoplastic CD34^+^ cells [127].

Collectively the reported studies show that the leukemia niche, in terms of LCSs and MSCs, is able to deregulate normal HSCs and neoplastic cells by EV-mediated communication.

### 5.2. HM Niche EVs versus MSCs

In the tumor context, MSC-EVs have a controversial role: they can promote or inhibit the tumor progression. These opposite effects of MSC-EVs can likely depend from both MSC source and culture conditions [128–130].

In general, EVs from healthy cells have a beneficial effect on recipient cells; on the contrary, EVs from cancer cells, have a detrimental influence also on MSCs [131]. MSCs exposed to tumor EVs acquire a series of functions such as migration to the tumor site [54, 132], production of proinflammatory cytokines [133], induction of prometastatic niches [134, 135], promotion of tumor growth *in vivo* [130], epithelial-to-mesenchymal transition induction [136, 137], recruitment of neoplastic cells in the BM [138], improvement of angiogenesis [139, 140], and modulation of the immune system [141–143].

Intriguingly, the crosstalk between tumor cells and MSCs seems to occur with a certain sequence: tumor cells, through EVs, communicate and modify MSCs; these reprogrammed MSCs, in response, produce EVs that can return on cancer cells or other cells creating a favorable environment for tumor [144, 145].

In HMs, less is known about the effect of neoplastic EVs on MSCs.

In CLL, Ghosh et al. found that MVs play an important role in the activation of the microenvironment in favor of disease progression [146]. CLL-MVs can activate the AKT signaling pathway in BM-MSCs by inducing the production of vascular endothelial growth factor, an important element for CLL cell survival [147]. In addition, Paggetti et al. demonstrated that CLL-derived Exo induce an inflammatory phenotype in endothelial cells and MSCs resembling the phenotype of cancer-associated fibroblasts [148]. In this way, leukemic Exo create a favorable environment for promoting CLL progression.

Exo derived from adult T-cell leukemia/lymphoma cells induce changes in cellular morphology and promote proliferation in MSCs by transferring epigenetic regulators, like *miRNA21* and *miRNA155* [149].

Horiguchi et al. found that EV *miRNA7977* derived from AML/MDS CD34^+^ cells, is transferred into BM-MSCs where it reduces the poly binding protein 1 levels by compromising their ability to support CD34^+^ cells [150]. Huan et al. studied the role of Exo in developing the BM AML niche [151]. They reported that leukemic Exo are taken up by BM stroma. These Exo deliver important AML pathogenesis mRNA such as FLT3, NPM1, IGF-IR, and CXCR4. In addition, they carry *miRNA150* which binds the receptor for SDF-1 and CXCR4 mRNA. Consequently, these Exo reduce the expression of CXCR4 and thus cell migration versus SDF-1 of target cells. The CXCR4/SDF-1 axis is fundamental for HSPC retention in BM and their differentiation. The last AML-Exo effects are an altered proliferation and migration of BM-MSCs and hematopoietic progenitor cell lines, by reprogramming the BM microenvironment [151].

Recently, Kumar et al. showed that, in *in vitro* and *in vivo* models, AML-Exo are internalized by BM cells, increase long-term HSC population, and alter stromal compartment [152]. They induce the osteoblast inhibitor DKK1 expression in MSC progenitors decreasing their osteoblast differentiation potential. AML-EVs reduce the expression of factors that support normal hematopoiesis such as CXCL12, KITL, and IL-7 in MSCs. These modified stromal cells enhance leukemia growth at the expense of normal hematopoiesis [152]. In another context, Exo released by CML cells stimulate BM-MSCs to produce IL-8, which, in turn, promote both *in vitro* and *in vivo* leukemic cell survival [153]. MVs containing “leukemic” transcripts from CML cells transfer these mRNAs in healthy MSCs, increasing their proliferation [108]. Finally, *miRNA146a* in EVs from MM cells is transferred in MSCs inducing the secretion of elevated levels of cytokines which improved both MM cell viability and migration [154].

Collectively, the reported data demonstrate that EVs derived from HM cells are efficiently transferred into MSCs to transform the BM microenvironment into a niche that supports malignancy at the expense of HSCs, although the mode of transformation is still uncertain.

## 6. Conclusions

In conclusion, EVs constitute a new bidirectional communication system between BM microenvironment and SCs. In fact, SCs, including HSCs and MSCs, are not only EV donors but also targets of EVs derived from BM cells. Specifically, immune cells communicate with MSCs via EVs modulating their phenotype. In addition, EVs represent a tool in mediating the MSC capacity to support HSPCs, improving their survival and clonogenic potential in physiological conditions.

In different HMs, EVs are significantly induced compared to healthy controls. Neoplastic EVs exert oncogenic functions that (1) boost malignancy through autocrine signaling, (2) induce a suppression of HSPC functions and SC malignant transformation, (3) modify the BM environment in favor of cancer/leukemic cells acting also on MSCs. These last cells exposed to tumor EVs acquire a series of functions such as migration to the tumor site, production of proinflammatory cytokines, induction of prometastatic niches, promotion of tumor growth *in vivo*, recruitment of neoplastic cells in the BM, improvement of angiogenesis, and modulation of the immune system. Overall, “tumor modified” MSCs release EVs that play an active role in supporting a favorable environment for malignant cells at the expense of normal hematopoiesis.

In order to render more transparent the field of EVs, an EV-TRACK platform is created to collect biological and technical information of EVs [155]. Further studies are needed to clarify not only the mechanism of action of EVs in disease and health, but also to define EV population-specific identity and cell origin, and the standardization of protocols for their isolation and characterization.

## References

[1] Eaves C. J. (2015). Hematopoietic stem cells: concepts, definitions, and the new reality. *Blood*.

[2] Morrison S. J., Scadden D. T. (2014). The bone marrow niche for haematopoietic stem cells. *Nature*.

[3] Sánchez-Aguilera A., Méndez-Ferrer S. (2017). The hematopoietic stem-cell niche in health and leukemia. *Cellular and Molecular Life Sciences*.

[4] Schepers K., Campbell T. B., Passegué E. (2015). Normal and leukemic stem cell niches: insights and therapeutic opportunities. *Cell Stem Cell*.

[5] Griessinger E., Anjos-Afonso F., Pizzitola I. (2014). A niche-like culture system allowing the maintenance of primary human acute myeloid leukemia-initiating cells: a new tool to decipher their chemoresistance and self-renewal mechanisms. *Stem Cells Translational Medicine*.

[6] Bonnet D., Dick J. E. (1997). Human acute myeloid leukemia is organized as a hierarchy that originates from a primitive hematopoietic cell. *Nature Medicine*.

[7] Zagozdzon R., Golab J. (2015). Review. Cancer stem cells in haematological malignancies. *Współczesna Onkologia*.

[8] Lawson C., Kovacs D., Finding E., Ulfelder E., Luis-Fuentes V. (2017). Extracellular vesicles: evolutionarily conserved mediators of intercellular communication. *Yale Journal of Biology and Medicine*.

[9] Ruivo C. F., Adem B., Silva M., Melo S. A. (2017). The biology of cancer exosomes: insights and new perspectives. *Cancer Research*.

[10] De Luca L., D’Arena G., Simeon V. (2017). Characterization and prognostic relevance of circulating microvesicles in chronic lymphocytic leukemia. *Leukemia & Lymphoma*.

[11] Whiteside T. L. (2017). Extracellular vesicles isolation and their biomarker potential: are we ready for testing?. *Annals of Translational Medicine*.

[12] Han L., Xu J., Xu Q., Zhang B., Lam E. W.-F., Sun Y. (2017). Extracellular vesicles in the tumor microenvironment: therapeutic resistance, clinical biomarkers, and targeting strategies. *Medicinal Research Reviews*.

[13] Nawaz M., Fatima F. (2017). Extracellular vesicles, tunneling nanotubes, and cellular interplay: synergies and missing links. *Frontiers in Molecular Biosciences*.

[14] Yuana Y., Sturk A., Nieuwland R. (2013). Extracellular vesicles in physiological and pathological conditions. *Blood Reviews*.

[15] Becker A., Thakur B. K., Weiss J. M., Kim H. S., Peinado H., Lyden D. (2016). Extracellular vesicles in cancer: cell-to-cell mediators of metastasis. *Cancer Cell*.

[16] Tkach M., Théry C. (2016). Communication by extracellular vesicles: where we are and where we need to go. *Cell*.

[17] Zhao H., Achreja A., Iessi E. (2018). The key role of extracellular vesicles in the metastatic process. *Biochimica et Biophysica Acta (BBA) - Reviews on Cancer*.

[18] Yáñez-Mó M., Siljander P. R.-M., Andreu Z. (2015). Biological properties of extracellular vesicles and their physiological functions. *Journal of Extracellular Vesicles*.

[19] Cappello F., Logozzi M., Campanella C. (2017). Exosome levels in human body fluids: a tumor marker by themselves?. *European Journal of Pharmaceutical Sciences*.

[20] Caivano A., Laurenzana I., De Luca L. (2015). High serum levels of extracellular vesicles expressing malignancy-related markers are released in patients with various types of hematological neoplastic disorders. *Tumor Biology*.

[21] Caivano A., La Rocca F., Simeon V. (2017). MicroRNA-155 in serum-derived extracellular vesicles as a potential biomarker for hematologic malignancies—a short report. *Cellular Oncology*.

[22] Cerulo L., Tagliaferri D., Marotta P. (2014). Identification of a novel gene signature of ES cells self-renewal fluctuation through system-wide analysis. *PLoS One*.

[23] Tagliaferri D., De Angelis M. T., Russo N. A. (2016). Retinoic acid specifically enhances embryonic stem cell metastate marked by Zscan4. *PLoS One*.

[24] Nawaz M., Fatima F., Vallabhaneni K. C. (2016). Extracellular vesicles: evolving factors in stem cell biology. *Stem Cells International*.

[25] Chen Z., Li Y., Yu H. (2017). Isolation of extracellular vesicles from stem cells. *Methods in Molecular Biology*.

[26] Ratajczak J., Miekus K., Kucia M. (2006). Embryonic stem cell-derived microvesicles reprogram hematopoietic progenitors: evidence for horizontal transfer of mRNA and protein delivery. *Leukemia*.

[27] Salvucci O., Jiang K., Gasperini P. (2012). MicroRNA126 contributes to granulocyte colony-stimulating factor-induced hematopoietic progenitor cell mobilization by reducing the expression of vascular cell adhesion molecule 1. *Haematologica*.

[28] Desrochers L. M., Antonyak M. A., Cerione R. A. (2016). Extracellular vesicles: satellites of information transfer in cancer and stem cell biology. *Developmental Cell*.

[29] Burrello J., Monticone S., Gai C., Gomez Y., Kholia S., Camussi G. (2016). Stem cell-derived extracellular vesicles and immune-modulation. *Frontiers in Cell and Development Biology*.

[30] Kordelas L., Rebmann V., Ludwig A.-K. (2014). MSC-derived exosomes: a novel tool to treat therapy-refractory graft-versus-host disease. *Leukemia*.

[31] Nassar W., El-Ansary M., Sabry D. (2016). Umbilical cord mesenchymal stem cells derived extracellular vesicles can safely ameliorate the progression of chronic kidney diseases. *Biomaterials Research*.

[32] Seita J., Weissman I. L. (2010). Hematopoietic stem cell: self-renewal versus differentiation. *Wiley Interdisciplinary Reviews: Systems Biology and Medicine*.

[33] Calvi L. M., Link D. C. (2015). The hematopoietic stem cell niche in homeostasis and disease. *Blood*.

[34] Lapidot T., Sirard C., Vormoor J. (1994). A cell initiating human acute myeloid leukaemia after transplantation into SCID mice. *Nature*.

[35] Wang J. C. Y., Dick J. E. (2005). Cancer stem cells: lessons from leukemia. *Trends in Cell Biology*.

[36] Coulombel L., Eaves C., Kalousek D., Gupta C., Eaves A. (1985). Long-term marrow culture of cells from patients with acute myelogenous leukemia. Selection in favor of normal phenotypes in some but not all cases. *The Journal of Clinical Investigation*.

[37] Ailles L. E., Gerhard B., Hogge D. E. (1997). Detection and characterization of primitive malignant and normal progenitors in patients with acute myelogenous leukemia using long-term coculture with supportive feeder layers and cytokines. *Blood*.

[38] van Gosliga D., Schepers H., Rizo A., van der Kolk D., Vellenga E., Schuringa J. J. (2007). Establishing long-term cultures with self-renewing acute myeloid leukemia stem/progenitor cells. *Experimental Hematology*.

[39] Zöller M. (2015). CD44, Hyaluronan, the hematopoietic stem cell, and leukemia-initiating cells. *Frontiers in Immunology*.

[40] Wasnik S., Kantipudi S., Kirkland M. A., Pande G. (2016). Enhanced *ex vivo* expansion of human hematopoietic progenitors on native and spin coated acellular matrices prepared from bone marrow stromal cells. *Stem Cells International*.

[41] Walenda T., Bokermann G., Ventura Ferreira M. S. (2011). Synergistic effects of growth factors and mesenchymal stromal cells for expansion of hematopoietic stem and progenitor cells. *Experimental Hematology*.

[42] Isern J., Martín-Antonio B., Ghazanfari R. (2013). Self-renewing human bone marrow mesenspheres promote hematopoietic stem cell expansion. *Cell Reports*.

[43] Verfaillie C. (1992). Direct contact between human primitive hematopoietic progenitors and bone marrow stroma is not required for long-term in vitro hematopoiesis. *Blood*.

[44] Horwitz E. M., Le Blanc K., Dominici M. (2005). Clarification of the nomenclature for MSC: the International Society for Cellular Therapy position statement. *Cytotherapy*.

[45] Dominici M., Le Blanc K., Mueller I. (2006). Minimal criteria for defining multipotent mesenchymal stromal cells. The International Society for Cellular Therapy position statement. *Cytotherapy*.

[46] Murray I. R., Péault B. (2015). Q&A: mesenchymal stem cells—where do they come from and is it important?. *BMC Biology*.

[47] Wagner W., Wein F., Seckinger A. (2005). Comparative characteristics of mesenchymal stem cells from human bone marrow, adipose tissue, and umbilical cord blood. *Experimental Hematology*.

[48] Calvi L. M., Adams G. B., Weibrecht K. W. (2003). Osteoblastic cells regulate the haematopoietic stem cell niche. *Nature*.

[49] Arai F., Hirao A., Ohmura M. (2004). Tie2/Angiopoietin-1 signaling regulates hematopoietic stem cell quiescence in the bone marrow niche. *Cell*.

[50] Chandran P., Le Y., Li Y. (2015). Mesenchymal stromal cells from patients with acute myeloid leukemia have altered capacity to expand differentiated hematopoietic progenitors. *Leukemia Research*.

[51] Ito S., Barrett A. J., Dutra A. (2015). Long term maintenance of myeloid leukemic stem cells cultured with unrelated human mesenchymal stromal cells. *Stem Cell Research*.

[52] Zhao Q., Ren H., Han Z. (2016). Mesenchymal stem cells: immunomodulatory capability and clinical potential in immune diseases. *Journal of Cellular Immunotherapy*.

[53] Watt S. M., Gullo F., van der Garde M. (2013). The angiogenic properties of mesenchymal stem/stromal cells and their therapeutic potential. *British Medical Bulletin*.

[54] Anthony B. A., Link D. C. (2014). Regulation of hematopoietic stem cells by bone marrow stromal cells. *Trends in Immunology*.

[55] Kfoury Y., Scadden D. T. (2015). Mesenchymal cell contributions to the stem cell niche. *Cell Stem Cell*.

[56] Hoogduijn M. J., Dor F. J. M. F. (2011). Mesenchymal stem cells in transplantation and tissue regeneration. *Frontiers in Immunology*.

[57] Zhao K., Liu Q. (2016). The clinical application of mesenchymal stromal cells in hematopoietic stem cell transplantation. *Journal of Hematology & Oncology*.

[58] Caivano A., Del Vecchio L., Musto P. (2017). Do we need to distinguish exosomes from microvesicles in hematological malignancies?. *Leukemia*.

[59] Raposo G., Stoorvogel W. (2013). Extracellular vesicles: exosomes, microvesicles, and friends. *Journal of Cell Biology*.

[60] Farooqi A. A., Desai N. N., Qureshi M. Z. (2018). Exosome biogenesis, bioactivities and functions as new delivery systems of natural compounds. *Biotechnology Advances*.

[61] Hessvik N. P., Llorente A. (2018). Current knowledge on exosome biogenesis and release. *Cellular and Molecular Life Sciences*.

[62] Wang J., Faict S., Maes K. (2016). Extracellular vesicle cross-talk in the bone marrow microenvironment: implications in multiple myeloma. *Oncotarget*.

[63] Lynch C., Panagopoulou M., Gregory C. D. (2017). Extracellular vesicles arising from apoptotic cells in tumors: roles in cancer pathogenesis and potential clinical applications. *Frontiers in Immunology*.

[64] Zaborowski M. P., Balaj L., Breakefield X. O., Lai C. P. (2015). Extracellular vesicles: composition, biological relevance, and methods of study. *Bioscience*.

[65] Meehan B., Rak J., Di Vizio D. (2016). Oncosomes—large and small: what are they, where they came from?. *Journal of Extracellular Vesicles*.

[66] Guo W., Gao Y., Li N. (2017). Exosomes: new players in cancer. *Oncology Reports*.

[67] Falchi A. M., Sogos V., Saba F., Piras M., Congiu T., Piludu M. (2013). Astrocytes shed large membrane vesicles that contain mitochondria, lipid droplets and ATP. *Histochemistry and Cell Biology*.

[68] Griessinger E., Moschoi R., Biondani G., Peyron J.-F. (2017). Mitochondrial transfer in the leukemia microenvironment. *Trends in Cancer*.

[69] Puhka M., Takatalo M., Nordberg M.-E. (2017). Metabolomic profiling of extracellular vesicles and alternative normalization methods reveal enriched metabolites and strategies to study prostate cancer-related changes. *Theranostics*.

[70] Sullivan L. B. (2017). Extracellular vesicles: taking metabolism on the road. *Nature Chemical Biology*.

[71] Kim D.-K., Lee J., Kim S. R. (2015). EVpedia: a community web portal for extracellular vesicles research. *Bioinformatics*.

[72] Kalra H., Simpson R. J., Ji H. (2012). Vesiclepedia: a compendium for extracellular vesicles with continuous community annotation. *PLoS Biology*.

[73] Keerthikumar S., Chisanga D., Ariyaratne D. (2016). ExoCarta: a web-based compendium of exosomal cargo. *Journal of Molecular Biology*.

[74] Dozio V., Sanchez J.-C. (2017). Characterisation of extracellular vesicle-subsets derived from brain endothelial cells and analysis of their protein cargo modulation after TNF exposure. *Journal of Extracellular Vesicles*.

[75] Minciacchi V. R., Freeman M. R., Di Vizio D. (2015). Extracellular vesicles in cancer: exosomes, microvesicles and the emerging role of large oncosomes. *Seminars in Cell & Developmental Biology*.

[76] Vallabhaneni K. C., Penfornis P., Dhule S. (2015). Extracellular vesicles from bone marrow mesenchymal stem/stromal cells transport tumor regulatory microRNA, proteins, and metabolites. *Oncotarget*.

[77] Kim H.-S., Choi D.-Y., Yun S. J. (2012). Proteomic analysis of microvesicles derived from human mesenchymal stem cells. *Journal of Proteome Research*.

[78] Baglio S. R., Rooijers K., Koppers-Lalic D. (2015). Human bone marrow- and adipose-mesenchymal stem cells secrete exosomes enriched in distinctive miRNA and tRNA species. *Stem Cell Research & Therapy*.

[79] Collino F., Deregibus M. C., Bruno S. (2010). Microvesicles derived from adult human bone marrow and tissue specific mesenchymal stem cells shuttle selected pattern of miRNAs. *PLoS One*.

[80] Gopal S. K., Greening D. W., Rai A. (2016). Extracellular vesicles: their role in cancer biology and epithelial-mesenchymal transition. *Biochemical Journal*.

[81] Prada I., Meldolesi J. (2016). Binding and fusion of extracellular vesicles to the plasma membrane of their cell targets. *International Journal of Molecular Sciences*.

[82] Choi D., Lee T. H., Spinelli C., Chennakrishnaiah S., D’Asti E., Rak J. (2017). Extracellular vesicle communication pathways as regulatory targets of oncogenic transformation. *Seminars in Cell & Developmental Biology*.

[83] Hoshino A., Costa-Silva B., Shen T.-L. (2015). Tumour exosome integrins determine organotropic metastasis. *Nature*.

[84] Montecalvo A., Larregina A. T., Shufesky W. J. (2012). Mechanism of transfer of functional microRNAs between mouse dendritic cells via exosomes. *Blood*.

[85] Hazan-Halevy I., Rosenblum D., Weinstein S., Bairey O., Raanani P., Peer D. (2015). Cell-specific uptake of mantle cell lymphoma-derived exosomes by malignant and non-malignant B-lymphocytes. *Cancer Letters*.

[86] Grange C., Tapparo M., Bruno S. (2014). Biodistribution of mesenchymal stem cell-derived extracellular vesicles in a model of acute kidney injury monitored by optical imaging. *International Journal of Molecular Medicine*.

[87] Peinado H., Alečković M., Lavotshkin S. (2012). Melanoma exosomes educate bone marrow progenitor cells toward a pro-metastatic phenotype through MET. *Nature Medicine*.

[88] Parolini I., Federici C., Raggi C. (2009). Microenvironmental pH is a key factor for exosome traffic in tumor cells. *Journal of Biological Chemistry*.

[89] De Luca L., Trino S., Laurenzana I. (2016). MiRNAs and piRNAs from bone marrow mesenchymal stem cell extracellular vesicles induce cell survival and inhibit cell differentiation of cord blood hematopoietic stem cells: a new insight in transplantation. *Oncotarget*.

[90] Fatima F., Ekstrom K., Nazarenko I. (2017). Non-coding RNAs in mesenchymal stem cell-derived extracellular vesicles: deciphering regulatory roles in stem cell potency, inflammatory resolve, and tissue regeneration. *Frontiers in Genetics*.

[91] Börger V., Bremer M., Ferrer-Tur R. (2017). Mesenchymal stem/stromal cell-derived extracellular vesicles and their potential as novel immunomodulatory therapeutic agents. *International Journal of Molecular Sciences*.

[92] Dostert G., Mesure B., Menu P., Velot É. (2017). How do mesenchymal stem cells influence or are influenced by microenvironment through extracellular vesicles communication?. *Frontiers in Cell and Development Biology*.

[93] Takeda Y. S., Xu Q. (2015). Neuronal differentiation of human mesenchymal stem cells using exosomes derived from differentiating neuronal cells. *PLoS One*.

[94] Lozito T. P., Tuan R. S. (2014). Endothelial and cancer cells interact with mesenchymal stem cells *via* both microparticles and secreted factors. *Journal of Cellular and Molecular Medicine*.

[95] Kim Y. J., Kim H. K., Cho H. H., Bae Y. C., Suh K. T., Jung J. S. (2007). Direct comparison of human mesenchymal stem cells derived from adipose tissues and bone marrow in mediating neovascularization in response to vascular ischemia. *Cellular Physiology and Biochemistry*.

[96] Pricola K. L., Kuhn N. Z., Haleem-Smith H., Song Y., Tuan R. S. (2009). Interleukin-6 maintains bone marrow-derived mesenchymal stem cell stemness by an ERK1/2-dependent mechanism. *Journal of Cellular Biochemistry*.

[97] Chiabotto G., Bruno S., Collino F., Camussi G. (2016). Mesenchymal stromal cells epithelial transition induced by renal tubular cells-derived extracellular vesicles. *PLoS One*.

[98] Omar O. M., Granéli C., Ekström K. (2011). The stimulation of an osteogenic response by classical monocyte activation. *Biomaterials*.

[99] Ekström K., Omar O., Granéli C., Wang X., Vazirisani F., Thomsen P. (2013). Monocyte exosomes stimulate the osteogenic gene expression of mesenchymal stem cells. *PLoS One*.

[100] Jang W.-G., Kim E.-J., Kim D.-K. (2012). BMP2 protein regulates osteocalcin expression via Runx2-mediated *Atf6* gene transcription. *Journal of Biological Chemistry*.

[101] Ratajczak J., Wysoczynski M., Hayek F., Janowska-Wieczorek A., Ratajczak M. Z. (2006). Membrane-derived microvesicles: important and underappreciated mediators of cell-to-cell communication. *Leukemia*.

[102] Ekström K., Valadi H., Sjöstrand M. (2012). Characterization of mRNA and microRNA in human mast cell-derived exosomes and their transfer to other mast cells and blood CD34 progenitor cells. *Journal of Extracellular Vesicles*.

[103] Stik G., Crequit S., Petit L. (2017). Extracellular vesicles of stromal origin target and support hematopoietic stem and progenitor cells. *Journal of Cell Biology*.

[104] Piro G., Carbone C., Cataldo I. (2016). An FGFR3 autocrine loop sustains acquired resistance to trastuzumab in gastric cancer patients. *Clinical Cancer Research*.

[105] Caivano A., La Rocca F., Laurenzana I. (2017). Extracellular vesicles in hematological malignancies: from biology to therapy. *International Journal of Molecular Sciences*.

[106] Arendt B. K., Walters D. K., Wu X., Tschumper R. C., Jelinek D. F. (2014). Multiple myeloma cell-derived microvesicles are enriched in CD147 expression and enhance tumor cell proliferation. *Oncotarget*.

[107] Wu X.-H. Leukemia-derived exosomes induce paracrine and autocrine cell proliferation in pediatric ALL.

[108] Milani G., Lana T., Bresolin S. (2017). Expression profiling of circulating microvesicles reveals intercellular transmission of oncogenic pathways. *Molecular Cancer Research*.

[109] Wojtuszkiewicz A., Schuurhuis G. J., Kessler F. L. (2016). Exosomes secreted by apoptosis-resistant acute myeloid leukemia (AML) blasts harbor regulatory network proteins potentially involved in antagonism of apoptosis. *Molecular & Cellular Proteomics*.

[110] Bouvy C., Wannez A., Laloy J., Chatelain C., Dogné J.-M. (2017). Transfer of multidrug resistance among acute myeloid leukemia cells via extracellular vesicles and their microRNA cargo. *Leukemia Research*.

[111] Roccaro A. M., Sacco A., Maiso P. (2013). BM mesenchymal stromal cell-derived exosomes facilitate multiple myeloma progression. *The Journal of Clinical Investigation*.

[112] Raimondi L., De Luca A., Amodio N. (2015). Involvement of multiple myeloma cell-derived exosomes in osteoclast differentiation. *Oncotarget*.

[113] Viola S., Traer E., Huan J. (2016). Alterations in acute myeloid leukaemia bone marrow stromal cell exosome content coincide with gains in tyrosine kinase inhibitor resistance. *British Journal of Haematology*.

[114] Xie Y., Zhang H., Li W. (2010). Dendritic cells recruit T cell exosomes via exosomal LFA-1 leading to inhibition of CD8^+^ CTL responses through downregulation of peptide/MHC class I and Fas ligand-mediated cytotoxicity. *The Journal of Immunology*.

[115] Hedlund M., Nagaeva O., Kargl D., Baranov V., Mincheva-Nilsson L. (2011). Thermal- and oxidative stress causes enhanced release of NKG2D ligand-bearing immunosuppressive exosomes in leukemia/lymphoma T and B cells. *PLoS One*.

[116] Reiners K. S., Topolar D., Henke A. (2013). Soluble ligands for NK cell receptors promote evasion of chronic lymphocytic leukemia cells from NK cell anti-tumor activity. *Blood*.

[117] Hong C.-S., Muller L., Whiteside T. L., Boyiadzis M. (2014). Plasma exosomes as markers of therapeutic response in patients with acute myeloid leukemia. *Frontiers in Immunology*.

[118] Wang J., De Veirman K., Faict S. (2016). Multiple myeloma exosomes establish a favourable bone marrow microenvironment with enhanced angiogenesis and immunosuppression. *The Journal of Pathology*.

[119] Garg T. K., Gann J. I., Malaviarachchi P. A. (2066). Myeloma-derived exosomes and soluble factors suppress natural killer cell function.

[120] Hornick N. I., Doron B., Abdelhamed S. (2016). AML suppresses hematopoiesis by releasing exosomes that contain microRNAs targeting c-MYB. *Science Signaling*.

[121] Huan J., Hornick N. I., Goloviznina N. A. (2015). Coordinate regulation of residual bone marrow function by paracrine trafficking of AML exosomes. *Leukemia*.

[122] Razmkhah F., Soleimani M., Mehrabani D. (2017). Leukemia microvesicles affect healthy hematopoietic stem cells. *Tumor Biology*.

[123] Gu H., Chen C., Hao X. (2016). Sorting protein VPS33B regulates exosomal autocrine signaling to mediate hematopoiesis and leukemogenesis. *The Journal of Clinical Investigation*.

[124] Wang Y., Cheng Q., Liu J., Dong M. (2016). Leukemia stem cell-released microvesicles promote the survival and migration of myeloid leukemia cells and these effects can be inhibited by microRNA34a overexpression. *Stem Cells International*.

[125] Trino S., Lamorte D., Caivano A. (2018). MicroRNAs as new biomarkers for diagnosis and prognosis, and as potential therapeutic targets in acute myeloid leukemia. *International Journal of Molecular Sciences*.

[126] Muntión S., Ramos T. L., Diez-Campelo M. (2016). Microvesicles from mesenchymal stromal cells are involved in HPC-microenvironment crosstalk in myelodysplastic patients. *PLoS One*.

[127] Ramos T. L., Sánchez-Abarca L. I., Rosón B. Extracellular vesicles play an important role in intercellular communication between bone marrow stroma and hematopoietic progenitor cells in myeloproliferative neoplasms.

[128] Lopatina T., Gai C., Deregibus M. C., Kholia S., Camussi G. (2016). Cross talk between cancer and mesenchymal stem cells through extracellular vesicles carrying nucleic acids. *Frontiers in Oncology*.

[129] Zhang X., Tu H., Yang Y., Fang L., Wu Q., Li J. (2017). Mesenchymal stem cell-derived extracellular vesicles: roles in tumor growth, progression, and drug resistance. *Stem Cells International*.

[130] Zhu W., Huang L., Li Y. (2012). Exosomes derived from human bone marrow mesenchymal stem cells promote tumor growth *in vivo*. *Cancer Letters*.

[131] Lindoso R. S., Collino F., Camussi G. (2015). Extracellular vesicles derived from renal cancer stem cells induce a pro-tumorigenic phenotype in mesenchymal stromal cells. *Oncotarget*.

[132] Sánchez C. A., Andahur E. I., Valenzuela R. (2016). Exosomes from bulk and stem cells from human prostate cancer have a differential microRNA content that contributes cooperatively over local and pre-metastatic niche. *Oncotarget*.

[133] Lee K., Park H., Lim E. H., Lee K. W. (2011). Exosomes from breast cancer cells can convert adipose tissue-derived mesenchymal stem cells into myofibroblast-like cells. *International Journal of Oncology*.

[134] Luga V., Wrana J. L. (2013). Tumor-stroma interaction: revealing fibroblast-secreted exosomes as potent regulators of Wnt-planar cell polarity signaling in cancer metastasis. *Cancer Research*.

[135] McBride J. D., Rodriguez-Menocal L., Guzman W., Candanedo A., Garcia-Contreras M., Badiavas E. V. (2017). Bone marrow mesenchymal stem cell-derived CD63^+^ exosomes transport Wnt3a exteriorly and enhance dermal fibroblast proliferation, migration, and angiogenesis in vitro. *Stem Cells and Development*.

[136] Syn N., Wang L., Sethi G., Thiery J.-P., Goh B.-C. (2016). Exosome-mediated metastasis: from epithelial-mesenchymal transition to escape from immunosurveillance. *Trends in Pharmacological Sciences*.

[137] Shi S., Zhang Q., Xia Y. (2016). Mesenchymal stem cell-derived exosomes facilitate nasopharyngeal carcinoma progression. *American Journal of Cancer Research*.

[138] Narayanan R., Huang C.-C., Ravindran S. (2016). Hijacking the cellular mail: exosome mediated differentiation of mesenchymal stem cells. *Stem Cells International*.

[139] Gong M., Yu B., Wang J. (2017). Mesenchymal stem cells release exosomes that transfer miRNAs to endothelial cells and promote angiogenesis. *Oncotarget*.

[140] Anderson J. D., Johansson H. J., Graham C. S. (2016). Comprehensive proteomic analysis of mesenchymal stem cell exosomes reveals modulation of angiogenesis via nuclear factor-KappaB signaling. *Stem Cells*.

[141] Del Fattore A., Luciano R., Pascucci L. (2015). Immunoregulatory effects of mesenchymal stem cell-derived extracellular vesicles on T lymphocytes. *Cell Transplantation*.

[142] Conforti A., Scarsella M., Starc N. (2014). Microvescicles derived from mesenchymal stromal cells are not as effective as their cellular counterpart in the ability to modulate immune responses in vitro. *Stem Cells and Development*.

[143] Chen W., Huang Y., Han J. (2016). Immunomodulatory effects of mesenchymal stromal cells-derived exosome. *Immunologic Research*.

[144] Yang Y., Bucan V., Baehre H., Von Der Ohe J., Otte A., Hass R. (2015). Acquisition of new tumor cell properties by MSC-derived exosomes. *International Journal of Oncology*.

[145] Haga H., Yan I. K., Takahashi K., Wood J., Zubair A., Patel T. (2015). Tumour cell-derived extracellular vesicles interact with mesenchymal stem cells to modulate the microenvironment and enhance cholangiocarcinoma growth. *Journal of Extracellular Vesicles*.

[146] Ghosh A. K., Secreto C. R., Knox T. R., Ding W., Mukhopadhyay D., Kay N. E. (2010). Circulating microvesicles in B-cell chronic lymphocytic leukemia can stimulate marrow stromal cells: implications for disease progression. *Blood*.

[147] Boysen J., Nelson M., Magzoub G. (2017). Dynamics of microvesicle generation in B-cell chronic lymphocytic leukemia: implication in disease progression. *Leukemia*.

[148] Paggetti J., Haderk F., Seiffert M. (2015). Exosomes released by chronic lymphocytic leukemia cells induce the transition of stromal cells into cancer-associated fibroblasts. *Blood*.

[149] El-Saghir J., Nassar F., Tawil N., El-Sabban M. (2016). ATL-derived exosomes modulate mesenchymal stem cells: potential role in leukemia progression. *Retrovirology*.

[150] Horiguchi H., Kobune M., Kikuchi S. (2016). Extracellular vesicle miR-7977 is involved in hematopoietic dysfunction of mesenchymal stromal cells via poly(rC) binding protein 1 reduction in myeloid neoplasms. *Haematologica*.

[151] Huan J., Hornick N. I., Shurtleff M. J. (2013). RNA trafficking by acute myelogenous leukemia exosomes. *Cancer Research*.

[152] Kumar B., Garcia M., Weng L. (2017). Acute myeloid leukemia transforms the bone marrow niche into a leukemia-permissive microenvironment through exosome secretion. *Leukemia*.

[153] Corrado C., Raimondo S., Saieva L., Flugy A. M., De Leo G., Alessandro R. (2014). Exosome-mediated crosstalk between chronic myelogenous leukemia cells and human bone marrow stromal cells triggers an interleukin 8-dependent survival of leukemia cells. *Cancer Letters*.

[154] De Veirman K., Wang J., Xu S. (2016). Induction of miR-146a by multiple myeloma cells in mesenchymal stromal cells stimulates their pro-tumoral activity. *Cancer Letters*.

[155] EV-TRACK Consortium, Van Deun J., Mestdagh P. (2017). EV-TRACK: transparent reporting and centralizing knowledge in extracellular vesicle research. *Nature Methods*.

